# Relationship between Air Pollution and Hospital Admissions for Chronic Obstructive Pulmonary Disease in Changchun, China: A Season-Stratified Case-Cross Study

**DOI:** 10.1155/2021/3240785

**Published:** 2021-07-16

**Authors:** Ye Ju, Xinli Ma, Huibo Li, Shuang Liu, A. Liya, Xinrong Guo

**Affiliations:** ^1^Department of Nursing Care of the Second Hospital, Jilin University, Changchun City 130022, Jilin, China; ^2^School of Public Health, Jilin University, Changchun City 130021, Jilin, China; ^3^College of Nursing, Beihua University, Jilin City 132208, Jilin, China

## Abstract

**Background:**

This study aimed to explore the relationship between air pollution and hospital admissions for COPD in Changchun, a northeast city of China, in different seasons.

**Methods:**

The data on a total of 1,733 hospitalized patients living in Changchun with acute exacerbation of COPD from September 2013 to April 2018 were collected from a comprehensive 3A hospital of Changchun. Daily average concentrations of PM2.5, PM10, SO_2_, NO_2_, CO, and O_3_ were collected from the Department of Ecology and Environment of Jilin Province. The conditional logistic regression model was adopted to analyze the effect of air pollutant concentration on the number of hospitalized patients with COPD in different seasons.

**Results:**

The maximum OR value for most air pollutants emitted in spring was on lag day 4, in summer and autumn on lag day 3, and in winter on lag day 2. In spring, SO_2_ and NO_2_ were entered into the regression equation, and the OR (95%CI) was 0.992 (0.986–0.998) and 1.009 (1.002–1.017); in autumn, PM2.5, PM10, and SO_2_ were entered into the regression equation, and the OR (95%CI) was 1.005 (1.000–1.011), 0.995 (0.991–1.000), and 1.006 (1.001–1.011), respectively; and in winter, PM2.5 and PM10 were entered into the regression equation, and the OR (95%CI) was 1.008 (1.002–1.015) and 0.994 (0.988–0.999), respectively.

**Conclusion:**

The relationship between air pollution and hospital admission for COPD in Northeast China varies with different seasons. In spring, NO_2_ is likely to be the major risk factor for hospital admissions for COPD; in autumn, PM2.5 and SO_2_ are the major risk factors; and in winter, PM2.5 is the major risk factor.

## 1. Introduction

In the past two decades, chronic obstructive pulmonary disease (COPD) has been a major cause of global mortality and morbidity and has produced an enormous burden on patients and the healthcare system [1, 2]. COPD is a common chronic respiratory disease which is closely related to environmental factors [3]. Previous research has suggested that the mortality risk of COPD posed by ambient air pollution was estimated to range from 1% to 21% [4]. Air pollutants which are able to enter into a person's airway include ozone (O_3_), carbon monoxide (CO), sulfur dioxide (SO_2_), nitrogen oxides (NO_2_), and suspended particles or particulate matter (PM) [5]. Larger particulate matter (PM) can deposit in the upper airway, while smaller PM can reach the alveoli and enter into the bloodstream [5, 6]. Several reports indicate that air pollutants, such as SO_2_, NO_2_, O_3_, and particulate matters, are causally associated with the development of COPD [7–9].

Hospital admission for COPD varies in different areas and in different seasons [10]. Changchun city is located in the northeastern part of China; it is part of eastern Eurasia and adjacent to the subfrigid zone. It is vulnerable to the northeast cold vortex in winter with frequent cold air flows which often produce adverse impacts on human health and result in meteorological diseases [8]. An acute attack of respiratory diseases is closely related to the surrounding environment and climatic conditions [8].

However, few studies have reported the association of air pollution with hospital admissions for COPD in the northeast of China. Moreover, the previous studies did not consider the seasonal conditions nor did they perform a stratified analysis. In addition, as a common chronic respiratory disease, it is difficult to determine the onset time of COPD. When acute exacerbation of COPD (AECOPD) occurs, patients will be sent to the hospital immediately, which can often indicate the onset time of AECOPD. Therefore, only newly diagnosed and admitted patients with AECOPD were included in the present study to determine the relationship between seasonal air pollutants with subsequent admissions to the hospital for AECOPD. Besides, a conditional logistic regression model, a more appropriate analysis method, was adopted to explore the relationship between air pollution and hospital admissions for AECOPD in Changchun in different seasons. This study is of great significance for the prevention and control of the acute aggravation of COPD caused by air pollution in different seasons in cold areas.

In China, the incidence of chronic obstructive pulmonary disease (COPD) is higher in the northeastern area than in other areas. This study aims to explore the relationship between air pollution and hospital admissions for COPD in Changchun, a northeast city of China, during different seasons since few studies are available about this specific relationship.

## 2. Materials and Methods

### 2.1. Data on Hospitalized COPD Patients

The data on a total of 1,733 hospitalized patients living in Changchun with acute exacerbation of COPD (Disease code: J44.51) (AECOPD) from September 2013 to April 2018 were collected from a comprehensive 3A hospital in Changchun, China. The data on COPD from this hospital are representative of the status of hospital admission for COPD in Changchun since the Department of Respiratory Diseases of this hospital is a National Key Discipline and has the largest number of hospital visits for COPD each year and an admittance rate of over 40% of the COPD cases in Changchun. COPD was diagnosed under the standards of the American Thoracic Society (ATS) with postbronchodilator FEV1/FVC <70% [11]. Patients admitted with the diagnosis of flu, bronchial asthma, or with other active pulmonary inflammatory diseases were excluded.

General information on the patients was also collected, including sex, age, home address, type of work, date of admission, admitting diagnosis, and medical history, and diagnosis, in accordance with the criteria for COPD clinical corresponding to diagnosis indicators.

This study was approved by the Institutional Review Board of the Second Hospital of Jilin University, Changchun, China.

### 2.2. Data on Air Pollutants

Data on the concentration of air pollutants at different monitoring sites in Changchun from September 2013 to April 2018 were obtained from the website of Ecology and Environment Department of Jilin Province. The data mainly cover six pollutants: PM_2.5_, PM_10_, SO_2_, NO_2_, O_3_, and CO.

### 2.3. Statistical Methods

A year was divided into spring (March to May), summer (June to August), autumn (September to November), and winter (December to February).

IBM SPSS 24.0 was used to analyze the data. The conditional logistic regression model was adopted to analyze the effect of air pollutant concentration on the number of newly diagnosed and admitted patients with COPD in different seasons. Multifactorial models assessing the relationship between hospital admissions of COPD and concentrations of air pollutants were based on the maximum lag effect in different seasons. Meteorological factors were included in the regression model to adjust the confounding effect.

## 3. Results

### 3.1. Characteristics of the Patients with COPD

The data of a total of 1733 AECOPD patients living in Changchun, China, were collected for the study. As shown in Table 1, the mean age of these patients was 69.94 ± 10.32 years. Most patients were female (55.7%). The seasonal distribution of AECOPD patients is autumn > winter > spring > summer.

### 3.2. Lag Effect of Different Pollutants in Different Seasons

As shown in Table 2, in spring, the maximum OR value for most air pollutants occurred on lag day 4. In summer, the maximum OR value occurred on lag day 3. In autumn, the maximum OR value also occurred on lag day 3. In winter, the maximum OR value occurred on lag day 2.

### 3.3. The Relationship between Hospital Admission for COPD and Concentration of Air Pollutants in Different Seasons

Based on the results in Table 2, lag day 4 in spring, lag day 3 in summer and autumn, and lag day 2 in winter were chosen as the exposure time. To control for the potential confounding by the day of the week, the seven days before exposure time were chosen as the control period.

As shown in Table 3, the results of multifactorial models showed that, after adjusting the meteorological factor, in spring, SO_2_ and NO_2_ were entered into the regression equation, and the OR (95%CI) was 0.992 (0.986–0.998) and 1.009 (1.002–1.017); in autumn, PM_2.5_, PM_10_, and SO_2_ were entered into the regression equation, and the OR (95%CI) was 1.005 (1.000–1.011), 0.995 (0.991–1.000), and 1.006 (1.001–1.011), respectively; and in winter, PM_2.5_ and PM_10_ were entered into the regression equation, and the OR (95%CI) was 1.008 (1.002–1.015) and 0.994 (0.988–0.999), respectively. There was not a significant effect of air pollutants on hospital admissions of AECOPD.

## 4. Discussion

In recent years, many studies have revealed that exposure to air pollutants can produce adverse impacts on people's health [12]. Air pollution is now fully acknowledged to be a significant public health problem and responsible for a growing range of health detriment, especially to the respiratory and circulatory systems [13, 14], in many regions of the world [15]. It is estimated that air pollution has been associated with the premature death of up to 3.7 million worldwide in 2012, with a significant proportion of these death in Asia (mainly in China and India) [16].

As the capital city of Jilin Province, China, the primary energy source for heating in winter in Changchun is mainly derived from coal. As the largest automobile industrial city in China, the vehicle ownership is increasing year by year, resulting in multiple sources of air pollution with the main source from coal combustion and the secondary source from on road vehicle usage. Due to the extended winter season and low temperatures in winter, the period of time for combustion of coal for indoor heating is more than five months, which causes a decrease in the air quality and produces a large number of pollutants [17]. Therefore, it is of great importance to explore the relationship between air pollution and respiratory diseases in Changchun, China.

As a common respiratory disease, COPD has been proven to be related to air pollution [8]. Only newly diagnosed and admitted patients with AECOPD were included in the present study to ensure the consistency of hospital visit and onset of COPD, which resulted in the relatively small sample size. According to our division of a year, autumn is from September to November; however, the heating in Changchun actually begins in October due to low temperature. Moreover, in October, the weather is very unpredictable and changes drastically, which will trigger an attack of AECOPD. Therefore, there are more AECOPD cases in autumn (33.9%) than in winter.

To better elucidate the effect of air pollution on hospital admissions for AECOPD, a case-cross study was used for this study [18]. The objects of the case-cross study include two parts, case and control; also, the information for both parts was obtained from the same individual. Therefore, the application of the case-cross study can fully control the influence of individual patient differences (such as sex, age, home address, and type of work) on the outcomes [18].

The climatic conditions and air pollution status vary in different seasons in Changchun city. We conducted a season-stratified case-cross study to analyze the relationship between air pollution and hospital admission for COPD in Changchun, China. Our findings suggest that exposure to air pollutants presents a lag effect on hospital admission for AECOPD. The lag effect for different seasons varies, and the lag days for different seasons are the days prior to the event of AECOPD; thus, the lag days were used as the exposure period for different seasons. To control for the potential confounding by the day of the week, the seven days before exposure time were chosen as the control period. Besides, we did a stratified analysis by season and adjusted the confounding factor of meteorological factor in our model.

The findings of this study on the effects of different seasons on COPD in the multifactorial models showed that the relationship between air pollution and hospital admission for COPD in Northeast China varies with the different seasons: in spring, for every 1 *μ*g/m^3^ increase in NO_2_ concentration, the number of inpatients with AECOPD increased by 0.9% (OR = 1.009, 95%CI = 1.002∼1.017), while in autumn, for every 1 *μ*g/m^3^ increase in SO_2_ concentration, the number of inpatients with AECOPD increased by 0.6% (OR = 1.001, 95%CI = 1.001∼1.011) and for every 1 *μ*g/m^3^ increase in PM_2.5_ concentration, the number of inpatients with acute COPD increased by 0.5%. Finally, in winter, for every 1 *μ*g/m^3^ increase in PM_2.5_ concentration, the number of inpatients increased by 0.8% (OR = 1.008, 95%CI = 1.002∼1.015).

Several studies examined the seasonal differences of the effects of NO_2_ and SO_2_ on diseases with inconsistent results [19–21]. The combined effects of high concentration of air pollutants and temperature levels, as well as ventilation conditions in different seasons, may explain the seasonal differences [22].

Air pollutants, such as CO, NO_2_, and O_3_, contributed to AECOPD in the warm season [23]. The reason may be that NO_2_ and O_3_ could trigger an inflammatory response, increasing the level of IL-8 [24–26]. IL-8 is a potent neutrophil chemoattractant [27], which could powerfully stimulate the secretion of mucin [28]. Increased systemic and sputum IL-8 concentrations have been associated with AECOPD [29, 30].

Due to the geographic location of Changchun, the winter lasts relatively long; thus, there is a longer heating period from October to April. During the heating period, the air quality is comparatively poor. Although the sources of air pollution in the mega cities have gradually changed from traditional coal combustion to a mix of coal combustion and vehicle emissions [31], also biomass burning in northeastern China contributes tremendously to the severe air pollution [32]. Exposure to oxidants and particles leads to exacerbation of inflammatory response which increases the number of hospitalizations due to infection [33, 34].

PM_2.5_ emission intensity varies with seasonal transition, with the highest concentration in winter and the lowest in summer. The main cause of this phenomenon is that, in winter, the inversion layer makes pollutants spread out not easily, while in summer, strong air convection makes pollutants dilute easily. Moreover, rain and wind facilitate the spread of pollutants, which explains the reason for the increased number of hospitalized patients with an acute aggravating period of COPD in winter. In addition, the high density of traffic and large industries located around the city also contribute to this phenomenon.

## 5. Conclusions

The distinctive four seasons cause the relationship between air pollutants and COPD in Changchun to vary. In spring, NO_2_ is likely to be a risk factor for cases of hospital admission for COPD; for every 1 *μ*g/m^3^ increase in NO_2_ concentration, the number of hospitalized patients with acute COPD increased by 0.9%. In autumn, PM_2.5_ and SO_2_ are identified as the risk factors; for every 1 *μ*g/m^3^ increase in PM_2.5_ concentration, the number of inpatients with COPD increased by 0.5%, while for every 1 *μ*g/m^3^ increase in SO_2_ concentration, the number of inpatients increased by 0.6%. In winter, PM_2.5_ was the main air pollutant producing more hospitalized patients with COPD; for every 1 *μ*g/m^3^ increase in PM_2.5_ concentration, the number of inpatients with acute COPD increased by 0.8%.

## Figures and Tables

**Table 1:** Characteristics of patients with COPD.

Characteristics	COPD (*N* = 1733)
*Sex*	
Male	768 (44.3%)
Female	965 (55.7%)
*Age*	69.94 ± 10.32
*Season of acute exacerbation*	
Spring	480 (27.7%)
Summer	152 (8.8%)
Autumn	587 (33.9%)
Winter	514 (29.6%)

**Table 2:** Hospital admissions for COPD patients and concentrations of air pollutants change on different lag days in different seasons.

Season	Lag days	PM_2.5_	PM_10_	SO_2_	NO_2_	CO	O_3_
		OR (95%CI)	OR (95%CI)	OR (95%CI)	OR (95%CI)	OR (95%CI)	OR (95%CI)
Spring	0	0.997 (0.992–1.001)	0.999 (0.998–1.001)	0.998 (0.986–1.011)	0.993 (0.984–1.002)	0.607 (0.376–0.981)	1.002 (0.997–1.006)
	1	1.002 (0.997–1.007)	1.000 (0.999–1.002)	1.000 (0.987–1.014)	1.003 (0.993–1.012)	1.393 (0.813–2.386)	1.001 (0.996–1.007)
	2	0.998 (0.993–1.003)	1.000 (0.998–1.001)	0.991 (0.977–1.005)	0.995 (0.985–1.005)	0.786 (0.434–1.425)	0.999 (0.994–1.005)
	3	1.001 (0.996–1.006)	1.001 (0.999–1.002)	0.999 (0.986–1.013)	1.000 (0.990–1.010)	1.042 (0.587–1.851)	1.003 (0.997–1.008)
	4	1.002 (0.997–1.007)	1.000 (0.998–1.002)	1.004 (0.993–1.016)	1.007 (0.998–1.015)	1.291 (0.804–2.072)	1.000 (0.995–1.004)

Summer	0	0.996 (0.998–1.004)	0.999 (0.994–1.003)	1.013 (0.961–1.068)	0.998 (0.986–1.011)	0.832 (0.388–1.782)	0.997 (0.992–1.002)
	1	1.002 (0.992–1.011)	0.999 (0.994–1.004)	0.965 (0.907–1.026)	0.996 (0.981–1.011)	1.089 (0.446–2.658)	1.001 (0.994–1.008)
	2	0.994 (0.986–1.003)	0.999 (0.995–1.004)	1.002 (0.942–1.066)	1.000 (0.985–1.016)	0.860 (0.346–2.139)	0.996 (0.989–1.003)
	3	1.007 (1.000–1.015)	1.003 (0.998–1.008)	1.015 (0.957–1.076)	1.000 (0.986–1.015)	1.531 (0.612–3.829)	1.002 (0.996–1.009)
	4	0.990 (0.982–0.999)	0.996 (0.992–1.000)	0.961 (0.912–1.013)	0.997 (0.985–1.010)	0.574 (0.259–1.272)	0.997 (0.992–1.002)

Autumn	0	1.000 (0.998–1.002)	1.000 (0.998–1.002)	0.999 (0.988–1.010)	0.999 (0.992–1.007)	0.985 (0.742–1.307)	0.997 (0.991–1.003)
	1	1.000 (0.997–1.002)	1.000 (0.998–1.002)	0.997 (0.982–1.012)	0.998 (0.990–1.006)	0.958 (0.690–1.330)	1.001 (0.994–1.007)
	2	1.000 (0.998–1.002)	1.000 (0.998–1.002)	1.006 (0.991–1.021)	1.000 (0.991–1.009)	0.948 (0.682–1.316)	1.000 (0.993–1.007)
	3	1.002 (0.999–1.004)	1.001 (0.999–1.003)	1.000 (0.986–1.015)	1.004 (0.996–1.012)	1.246 (0.894–1.737)	0.999 (0.992–1.005)
	4	0.999 (0.997–1.002)	1.000 (0.998–1.001)	1.004 (0.992–1.016)	1.000 (0.993–1.007)	1.005 (0.780–1.294)	1.000 (0.994–1.005)

Winter	0	1.000 (0.998–1.003)	1.000 (0.998–1.002)	1.001 (0.995–1.006)	1.002 (0.994–1.009)	0.945 (0.772–1.237)	1.001 (0.991–1.011)
	1	0.999 (0.997–1.002)	1.000 (0.998–1.002)	1.001 (0.994–1.008)	0.998 (0.988–1.008)	1.022 (0.748–1.396)	1.007 (0.995–1.020)
	2	1.002 (0.999–1.005)	1.001 (0.998–1.003)	1.000 (0.993–1.007)	1.009 (0.998–1.020)	1.279 (0.909–1.801)	0.991 (0.977–1.005)
	3	0.998 (0.994–1.001)	0.999 (0.996–1.001)	0.998 (0.991–1.004)	0.992 (0.981–1.002)	0.783 (0.559–1.097)	1.005 (0.991–1.019)
	4	1.000 (0.997–1.003)	1.000 (0.998–1.002)	1.000 (0.994–1.005)	0.999 (0.991–1.007)	0.969 (0.747–1.257)	1.003 (0.992–1.014)

**Table 3:** Multifactorial models to assess the relationship between hospital admissions of COPD and concentrations of air pollutants in different seasons.

Season	Air pollutants	*B*	SE	Wald	*P*	OR (95%CI)^*∗*^
Spring	SO_2_	−0.008	0.003	7.077	0.008	0.992 (0.986–0.998)
	NO_2_	0.009	0.004	5.557	0.018	1.009 (1.002–1.017)

Autumn	PM_2.5_	0.005	0.003	4.362	0.037	1.005 (1.000–1.011)
	PM_10_	−0.005	0.002	4.302	0.038	0.995 (0.991–1.000)
	SO_2_	0.006	0.006	5.219	0.022	1.006 (1.001–1.011)

Winter	PM_2.5_	0.008	0.003	5.871	0.015	1.008 (1.002–1.015)
	PM_10_	−0.006	0.003	4.892	0.027	0.994 (0.988–0.999)

^*∗*^After adjusting the meteorological factor.

## Data Availability

Data are available on request. Contact information: guoxinrong1949@163.com.
